# Editorial for Special Issue “Linking Genomic Changes with Cancer in the NGS Era, 2nd Edition”

**DOI:** 10.3390/cimb47110937

**Published:** 2025-11-11

**Authors:** Javier Azúa-Romeo

**Affiliations:** 1 Department of Human Anatomy and Histology, Faculty of Medicine, University of Zaragoza, 50009 Zaragoza, Spain; jazua@analizalab.com; 2 Department of Pathology, Analiza, 28001 Madrid, Spain

Next-generation sequencing (NGS) has revolutionized our understanding of cancer by enabling the comprehensive genomic profiling of tumors. This technology reveals a spectrum of somatic mutations, structural variants, and epigenetic alterations that drive tumorigenesis, progression, and therapeutic resistance. By decoding the cancer genome with unprecedented detail, NGS has paved the way for precision oncology, facilitating the discovery of novel biomarkers and targeted therapies.

Key studies highlight the transformative impact of NGS. The Cancer Genome Atlas (TCGA) has defined molecular subtypes of glioblastoma, breast cancer, and lung adenocarcinoma, identifying actionable mutations in genes such as TP53, PIK3CA, and EGFR. In this regard, NGS offers significant advantages by identifying targetable genomic alterations and revealing new therapeutic targets. In tumors where treatment options are limited, such as sarcomas, this technology plays a crucial role [1].

Among its many applications, high-throughput sequencing (NGS) is essential for identifying common pathogenic somatic mutations associated with specific processes like colorectal carcinogenesis. In this context, NGS has contributed to characterizing these mechanisms by highlighting frequently mutated genes such as TP53 and APC, and identifying novel genetic alterations previously unreported in colorectal cancer patients [2]. Similarly, in gallbladder cancer (GBC), comprehensive genomic analysis using NGS enabled the identification of somatic mutations across multiple genes, as well as targetable genetic alterations in genes such as BRCA1/2, EGFR, and ERBB2 [3].

Regarding myxoid liposarcomas (MLS), these commonly harbor the FUS::DDIT3 gene fusion. However, in one particular case, although fluorescence in situ hybridization (FISH) did not detect rearrangements at the DDIT3 locus, next-generation sequencing identified a novel gene fusion between SMARCA2 and DDIT3. The tumor exhibited the classic histological features of MLS, and thanks to NGS, an atypical and complex rearrangement was detected, revealing a relevant molecular alteration not previously identified by conventional techniques [4].

Moreover, NGS has enabled the association of specific genetic variants with particular cancer types; for example, SOD2 gene variants rs4880 and rs5746136 have been linked to breast cancer risk [5]. Similarly, sequencing efforts in hematologic malignancies such as acute myeloid leukemia have uncovered recurrent mutations in FLT3, NPM1, and DNMT3A, improving prognosis and guiding therapeutic strategies.

This technology also allows for the detection of rare genetic alterations in certain tumor types. For instance, NGS facilitated the simultaneous detection of mutations in IDH1 and IDH2 within a single astrocytoma (glioma)—a co-occurrence rarely described in existing literature [6].

The integration of multi-omic data—including genomic, transcriptomic, and epigenomic information—has further illuminated the complexity and heterogeneity of cancer. For example, NGS studies in lung squamous cell carcinoma have linked distinct mutational signatures to smoking history, enabling personalized treatments. Additionally, liquid biopsies leveraging circulating tumor DNA (ctDNA) allow for non-invasive cancer monitoring, early detection, and assessment of minimal residual disease.

Despite these advances, challenges remain. The interpretation of vast genomic datasets requires advanced bioinformatics to distinguish driver mutations from passenger alterations. Intratumoral heterogeneity complicates target identification, necessitating spatial and temporal sequencing. Translating genomic insights into clinical practice also faces barriers such as cost, infrastructure, standardization, and regulatory hurdles.

Looking ahead, emerging NGS technologies—including long-read sequencing, single-cell genomics, and real-time sequencing—promise deeper insights into tumor biology. Artificial intelligence and machine learning will enhance biomarker discovery and predictive modeling, supporting personalized therapies. Furthermore, understanding tumor microenvironment interactions and epigenetic regulation will drive combination strategies to overcome therapeutic resistance.

In summary, NGS has profoundly transformed cancer genomics and precision medicine. The works included in this Special Issue exemplify the technological and conceptual advances that are reshaping the way we understand tumor biology and develop personalized therapeutic strategies. Continued integration of genomic data into clinical decision-making will be essential to fully realize the promise of precision oncology and improve outcomes for patients worldwide.

## References

[1] Gündoğdu Y., Şenocak Taşçı E., Özer L., Boynukara C., Çeçen R., Mutlu A.U., Yıldız İ. (2025). Next-generation sequencing-based genomic profiling of advanced soft tissue and bone sarcomas. Front. Oncol..

[2] Youssef A.S.E.-D., Abdel-Fattah M.A., Lotfy M.M., Nassar A., Abouelhoda M., Touny A.O., Hassan Z.K., Eldin M.M., Bahnassy A.A., Khaled H. (2022). Multigene Panel Sequencing Reveals Cancer-Specific and Common Somatic Mutations in Colorectal Cancer Patients: An Egyptian Experience. Curr. Issues Mol. Biol..

[3] Erices J.I., González E., Salgado M., Barahona-Ponce C., Freire M., Sepúlveda-Hermosilla G., Ampuero D., Blanco A., Gárate-Calderón V., Báez-Benavides P. (2025). The mutational landscape and actionable targets of gallbladder cancer: An ancestry-informed and comparative analysis of a Chilean population. Front. Oncol..

[4] Shah A., Sukov W.R., Halling K., Marcelis L., Oliveira A. (2025). Novel SMARCA2::DDIT3 Fusion in Primary Subcutaneous Myxoid Liposarcoma due to an Unusual Unbalanced Chromosomal Translocation. Genes Chromosomes Cancer.

[5] Gallegos-Arreola M.P., Ramírez-Patiño R., Sánchez-López J.Y., Zúñiga-González G.M., Figuera L.E., Delgado-Saucedo J.I., Gómez-Meda B.C., Rosales-Reynoso M.A., Puebla-Pérez A.M., Lemus-Varela M.L. (2022). SOD2 Gene Variants (rs4880 and rs5746136) and Their Association with Breast Cancer Risk. Curr. Issues Mol. Biol..

[6] Yuile A., Satgunaseelan L., Wei J., Kastelan M., Back M.F., Lee M., Wei H., Buckland M.E., Lee A., Wheeler H.R. (2022). Implications of Concurrent IDH1 and IDH2 Mutations on Survival in Glioma—A Case Report and Systematic Review. Curr. Issues Mol. Biol..

